# Nab-paclitaxel plus S-1 as first-line followed by S-1 maintenance for advanced pancreatic adenocarcinoma: a single-arm phase II trial

**DOI:** 10.1007/s00280-018-3650-4

**Published:** 2018-07-23

**Authors:** Wen Zhang, Chunxia Du, Yongkun Sun, Lin Yang, Chengxu Cui, Zhichao Jiang, Chengfeng Wang, Jinwang Wang, Aiping Zhou

**Affiliations:** 1 Department of Medical Oncology, National Cancer Center/National Clinical Research Center for Cancer/Cancer Hospital, Chinese Academy of Medical Science and Peking Union Medical College, Beijing, 100021 China; 2 Department of Pancreatic and Gastric Surgery, National Cancer Center/National Clinical Research Center for Cancer/Cancer Hospital, Chinese Academy of Medical Science and Peking Union Medical College, Beijing, 100021 China

**Keywords:** Nab-paclitaxel, S-1, Objective response rate, Overall survival, Advanced pancreatic adenocarcinoma

## Abstract

**Purpose:**

We conducted a single-arm prospective phase II study to determine the efficacy and safety of the first-line treatment of advanced pancreatic cancer with nab-paclitaxel and S-1 followed by S-1 maintenance therapy.

**Methods:**

Nab-paclitaxel was administered intravenously on days 1 and 8 at 120 mg/m^2^. S-1 at 120 mg/day (for surface area ≥ 1.5 m^2^), 100 mg/day (for surface area between 1.25–1.5 m^2^), and 80 mg/day (for surface area < 1.25 m^2^) were given two times daily on days 1–14 every 3 weeks. Patients who achieved response and stable disease after 6 cycles were given S-1 maintenance treatment in the same schedule until disease progression or unacceptable toxicity developed. The primary endpoint was objective response rate (ORR), and the secondary endpoints were disease control rate (DCR), progression-free survival (PFS), overall survival (OS) and safety. Between 01/2015 and 07/2017, 32 patients were enrolled.

**Results:**

The ORR in the intention-to-treat (ITT) population (*N* = 32) was 53.1%, and the DCR was 87.5%. In the 30 evaluable patients, the ORR and DCR were 56.7 and 93.3%, respectively. The median follow-up time was 18 (range 12–36) months, the median PFS was 6.2 (range 4.4–8) months, and the median OS was 13.6 (range 8.7–18.5) months. The incidence of grade 3/4 neutropenia was 27.6%. Other grade 3 adverse events included 1 (3.1%) hand–foot syndrome, 1 (3.1%) rash and 2 (6.3%) diarrheas.

**Conclusions:**

Nab-paclitaxel and S-1 regimen has presented encouraging ORR, OS, and manageable toxicities as first-line therapy for advanced pancreatic cancer.

## Instructions

Pancreatic cancer is one of the most fatal malignant disease worldwide with an increasing incidence. It is the fourth and seventh leading cause of cancer-related mortality in the world [1] and China [2] respectively according to 2014 statistics. The vast majority of patients are confirmed as locally advanced or metastatic disease at diagnosis with poor prognosis and an overall 5-year survival rate of approximately 4% [3]. Advanced pancreatic cancer is characterized by poorer prognosis.

Gemcitabine has been approved as the standard chemotherapy for advanced pancreatic cancer since 1996, but the efficacy is extremely limited with a response rate of 6–8% and median survival of 5.5–7 months. Gemcitabine-based combination either with 5-FU or oxaliplatin and irinotecan failed to gain overall survival benefit compared to gemcitabine alone [4, 5]. Gemcitabine had been the standard care for advanced pancreatic cancer for more than 15 years until 2011 the treatment of leucovorin, 5-fluorouracil, irinotecan, and oxaliplatin (FOLFIRINOX) was reported for better ORR, PFS, and OS [6], followed by the combination of nab-paclitaxel and gemcitabine reported in 2013. Though these two novel combination regimens have become the recommendation priority for advanced pancreatic cancer in NCCN guideline, the response rate remains 23–31% and the overall survival less than 1 year. Moreover, the triplet therapy of FOLFIRINOX were confirmed much more toxic than gemcitabine, resulting the limited application in the real world. Therefore, there is still a great need to explore more effective systemic regimen with favorable safety profile for advanced pancreatic cancer.

S-1, a new oral fluoropyrimidine derivative, has been approved for advanced pancreatic cancer in Japan [7, 8] and is widely used in other Asian countries. As a monotherapy, S1 achieved an overall response rate of approximately 20% in early studies. Both nab-paclitaxel and S-1 are the most effective drugs for advanced pancreatic cancer, but limited data are available on the efficacy and safety of nab-paclitaxel plus S-1 so far. Thus, we conducted a single-arm prospective phase II study to evaluate the efficacy and safety of such combination as the first-line treatment for advanced pancreatic cancer.

The study was approved by the Ethical Committee of Cancer Institute and Hospital, Chinese Academy of Medical Sciences, No.14–102/892.

## Information and methods

### Patients

Patients aged ≥ 18 years with histologically or cytologically confirmed pancreatic cancer were enrolled. Patients with locally advanced or metastatic disease, performance status 0–1, presence of at least one measurable lesion according to Response Evaluation Criteria in Solid Tumors (RECIST 1.1), no prior systemic chemotherapy, normal blood routine, normal liver and kidney functions, and roughly normal electrocardiograph index were recruited.

## Methods

Patients with locally advanced or metastatic pancreatic cancer were treated with nab-paclitaxel and S-1 as first-line therapy. Nab-paclitaxel was administered intravenously on days 1 and 8 at 120 mg/m^2^. S-1 at 120 mg/day for surface area ≥ 1.5 m^2^, 100 mg/day for surface area between 1.25 and 1.5 m^2^, and 80 mg/day for surface area < 1.25 m^2^ were given 2 times daily on days 1–14 every 3 weeks. S-1 maintenance treatment was given to patients who achieved response and stable disease after 6 cycles of therapy until disease progression or unacceptable toxicity developed. Routine blood work, liver and kidney function tests, and electrocardiogram examination were performed before treatment. Doses were reduced by 20–25% in the next cycle for any grade 4 hematologic toxicity and grade 3 non-hematologic toxicity (with the exception of nausea and hair loss).

### Evaluation of efficacy and safety

All patients were monitored by imaging examination every 2 cycles to evaluate the efficacy according to RECIST 1.1 [9] for complete response (CR), partial response (PR), stable disease (SD), and disease progression (PD). CR + PR was defined as objective response rate (ORR), and CR + PR + SD was defined as disease control rate (DCR). PFS was calculated from study entry to disease progression or death. OS was calculated from study entry to death for any cause. NCI-CTCAE 4.0 was used to assess adverse reactions.

The primary endpoint was objective response rate (ORR), and the second endpoints included disease control rate (DCR), progression-free survival (PFS), overall survival (OS) and safety.

### Statistics

SPSS 17.0 software was used for statistical analysis. Survival was analyzed using Kaplan–Meier method and log-rank test. Cox proportional hazard regression model was used to examine both univariate and multivariate associations with survival. The *χ*^2^ test used an *α* = 0.05.

## Results

32 patients were enrolled between 01/2015 and 07/2017. The median age was 53 years (range 37–70 years), including 21 males and 11 females. All patients had performance status of 0–1. The most common metastatic site was liver, accounting for 71.9%. The baseline characteristics are described in Table 1.

**Table 1:** Patient demographics and disease characteristics at baseline

Characteristics	*N* (%)
Age (years)	
Median (range)	53 (37–70)
≥ 65 years	6 (18.7%)
Gender	
Male	21 (65.6%)
Female	11(34.4%)
ECOG PS	
0	10 (31.3%)
1	22 (68.7%)
Pancreatic primary tumor location	
Head	15 (46.9%)
Body/tail	17 (53.1%)
Current site(s) of metastasis	
Lung	2 (6.3%)
Liver	23 (71.9%)
Peritoneum	5 (15.6%)
Lymph node	9 (28.1%)
Previous surgery	
Yes	6 (18.8%)
No	26 (81.2%)
CA19-9	
Normal	3 (9.4%)
Abnormal	29 (90.6%)
Median at diagnosis, U/ml (range)	432 (58.54–34354)
Number of metastatic site	
1	11 (34.4%)
≥ 2	21 (65.6%)

*ECOG PS* Eastern Cooperative Oncology Group Performance Status, *CA 19-1* carbohydrate antigen 19-9

### Efficacy

**Fig. 1:**
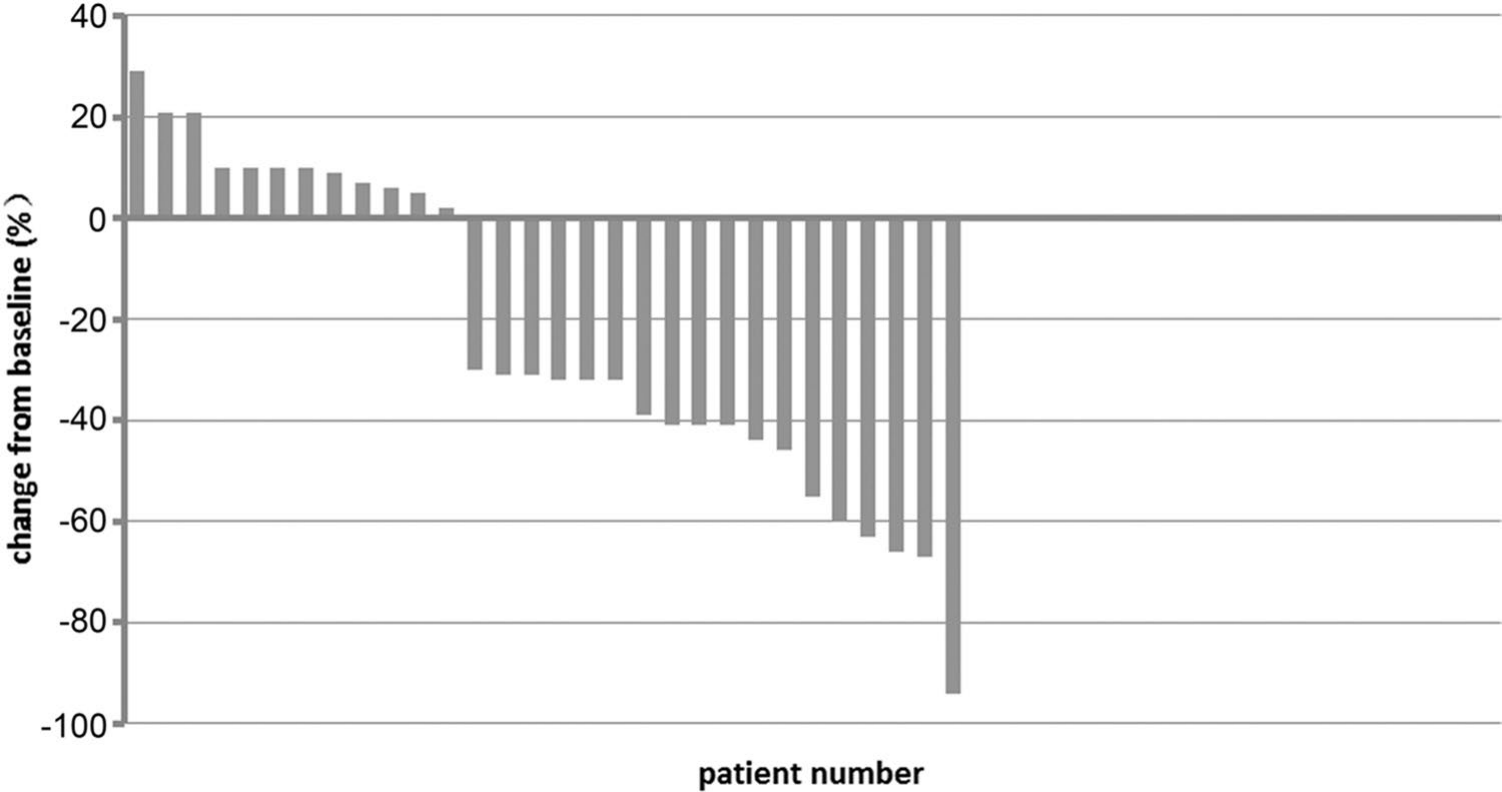
Waterfall plot. The best percentage change in target lesion determined by RECIST 1.1 for all evaluable patients (*N* = 30), and the dashed lines at 20 and − 30% represent progressive disease and partial response

32 patients received a median 6 (range 1–6) cycles. One patient withdrew from the study after 1 cycle of treatment due to abdominal pain. One carcinoma in pancreatic head with involvement of duodenum and hepatic metastasis withdrew and received palliative radiotherapy after 1 cycle of treatment for duodenal obstruction. The ORR in the intention-to-treat (ITT) population (*N* = 32) was 53.1% (Table 2), and the disease control rate (DCR) was 87.5%. The ORR and DCR were 56.7 and 93.3%, respectively, in the 30 evaluable patients. A waterfall plot of the best response based on independent imaging assessment was shown in Fig. 1, whereas the best response was defined as the best target lesion(s)- response recorded from the start to the end of treatment.

**Table 2:** ORR in patients treated with nab-paclitaxel plus S-1 (ITT population)

Best response (*n* = 32)	Number of patients, *n* (%)
CR	0
PR	17 (53.1)
SD	11 (34.4)
PD	2 (6.3)
Not evaluable^a^	2 (6.3)
DCR (CR + PR + SD)	28 (87.5)

*CR* complete response, *PR* partial response, *SD* stable disease, *PD* progressive disease, *DCR* disease control rate

^a^Two patients had no response evaluation, 1 patient withdrew from the study after 1 cycle of treatment due to abdominal pain, 1 carcinoma in pancreatic head with involvement of duodenum and hepatic metastasis withdrew and received palliative radiotherapy after 1 cycle of treatment for duodenal obstruction

In 29 patients (90.6%) with elevated carbohydrate antigen 19-9 (CA19-9) at baseline, 15 (52.3%) achieved ≥ 50% decline from baseline.

### Maintenance treatment

19 patients (59%) completed 6 cycles of planned treatment. 13 patients received S-1 maintenance treatment, in which 11 patients were treated for 6 cycles, while the other 2 patients had response but started maintenance treatment after 4 cycles due to patients’ preference or adverse event. The median time of maintenance treatment was 3 (range 1–6) months.

### Subsequent treatment

11 patients received gemcitabine plus oxaliplatin as the second-line treatment after failure of first-line chemotherapy, 2 PR (18.1%), 2 SD (18.1%), and 7 PD (63.6%) were reported. Irinotecan-based two drug combination regimen was given as the third-line chemotherapy for four patients, one had PR and three had PD.

### Survival analysis

**Fig. 2:**
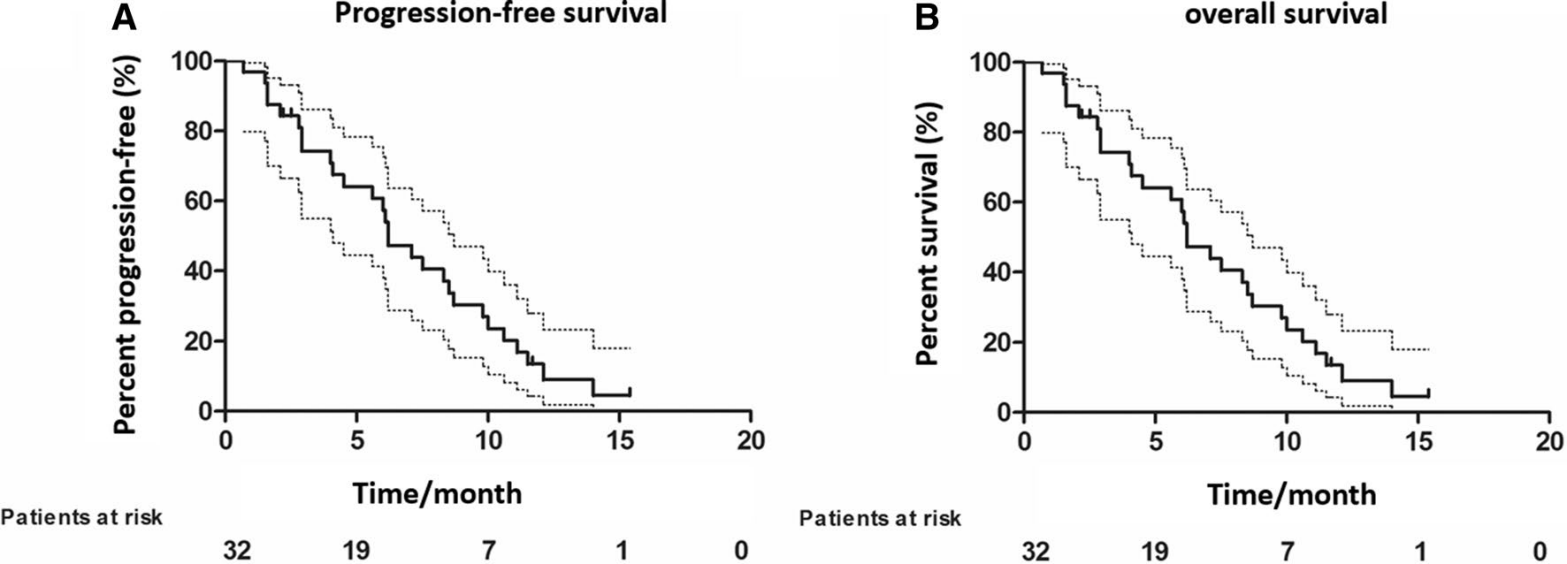
**a** Kaplan–Meier estimates of PFS in patients with mPC treated with nab-paclitaxel and S-1. PFS, progression-free survival; mPC, metastatic pancreatic adenocarcinoma. **b** Kaplan–Meier estimates of OS in patients with mPC treated with nab-paclitaxel and S-1. OS, overall survival; mPC, metastatic pancreatic adenocarcinoma

The median follow-up time was 18 (range 12–36) months, median PFS was 6.2 (range 4.4–8) months, and median OS was 13.6 (range 8.7–18.5) months, and 12 patients survived as of the last follow-up (Fig. 2a, b).

### Univariate and multivariate analysis

Univariate and multivariate analysis were performed for disease survival to adjust for performance status (0 vs.1), primary lesion (pancreatic head vs. tail), number of metastatic sites (1 vs. more), gender (male vs. female), age (≥ 60 vs. < 60), ≥ 50% decline from baseline CA19-9 (yes vs. no), with only liver metastasis (yes vs. no) by Cox regression model. No significant correlation was observed between the efficacy of the combination regimen and above clinical factors in the treatment.

### Adverse events

As shown in Table 3, 32 patients were evaluable for safety analysis. No treatment-related deaths occurred. The most common adverse events included grade 1–2 nausea (69%), anorexia (69%), neutropenia (55.2%), hair loss (37.9%), and anemia (34.5%). The incidence of grade 3/4 neutropenia was 27.6%. Other grade 3 adverse events included 1 hand–foot syndrome related to S-1 treatment, 1 rash and 2 diarrheas. The overall incidence of grade 3–4 adverse events was 41.5%. Dose reduction and adjustment because of adverse events occurred in 5 patients (15.6%).

**Table 3:** Adverse events

Adverse event	Incidence, *n* (%)			
	Grade 1	Grade 2	Grade 3	Grade 4
Neutropenia	8 (27.6)	8 (27.6)	4 (13.8)	4 (13.8)
Febrile neutropenia	1 (13.8)	0 (0.0)	0 (0.0)	0 (0.0)
Anemia	9 (31.03%)	1 (3.5%)	0 (0.0)	0 (0.0)
Thrombocytopenia	4 (13.8%)	3 (10.3%)	0 (0.0)	0 (0.0)
ALT elevation	1 (3.5%)	2 (6.9%)	0 (0.0)	0 (0.0)
TBIL elevation	2 (6.9%)	1 (3.5%)	0 (0.0)	0 (0.0)
Hair loss	9 (31.03%)	2 (6.9%)	0 (0.0)	0 (0.0)
Fatigue	3 (10.3%)	3 (10.3%)	0 (0.0)	0 (0.0)
Hand–foot syndrome	0 (0.0)	3 (10.3%)	1 (3.5%)	0 (0.0)
Peripheral neuropathy	4 (13.8%)	0 (0.0)	0 (0.0)	0 (0.0)
Anorexia	16 (55.2%)	4 (13.8%)	0 (0.0)	0 (0.0)
Nausea	14 (48.3%)	6 (20.7%)	0 (0.0)	0 (0.0)
Vomiting	5 (17.2%)	2 (6.9%)	0 (0.0)	0 (0.0)
Stomatitis	0 (0.0)	2 (6.9%)	0 (0.0)	0 (0.0)
Diarrhea	2 (6.9%)	1 (3.5%)	2 (6.9%)	0 (0.0)
Rash	2 (6.9%)	0 (0.0)	1 (3.5%)	0 (0.0)

## Discussion

Nab-paclitaxel and S-1 act on the M and S phases of the cell cycle, respectively. Masaya [10] found that the combination of S-1 and nab-paclitaxel had a synergetic effect in preclinical studies and might play a role in stromal depletion and tumor angiogenesis. This combination therapy may warrant further evaluation by clinical trials in pancreatic cancer patients.

In this study, nab-paclitaxel plus S-1 followed by S-1 maintenance therapy was proved an encouraging outcome as the first line treatment for advanced pancreatic cancer. The ORR and DCR reached 53.1 and 87.5%, higher than those of FOLFIRINOX, gemcitabine plus nab-paclitaxel (AG) and gemcitabine plus S-1(GS) in phase III trials. In the MPACT study [11], 861 patients with metastatic pancreatic cancer were randomized for AG and gemcitabine regimen. The ORR of AG was 23% assessed by the investigator, much higher than that of gemcitabine. In a phase II/III trail [6], 342 patients with advanced pancreatic cancer were randomized to receive either FOLFIRINOX or gemcitabine monotherapy. The ORR of FOLFIRINOX was 31.6%, compared to 9% of gemcitabine monotherapy (*P* = 0.0008), and has never been surpassed so far.

The survival in this study also seemed pretty encouraging. The PFS was 6.2 months and the OS exceeded 12 months in contrast to the median OS of 8.5 vs. 6.7 months (*P* = 0.000015) and the PFS of 5.5 vs. 3.7 months (*P* = 0.000024), in AG and gemcitabine group in MPACT trial. The PFS was 6.4 vs. 3.3 months (*P* < 0.0001), and the OS was 11.1 vs. 6.8 months (*P* < 0.001) in the FOLFIRINOX and GEM group, respectively [6].

GS is another widely used combination regimen in Asia. In the phase III study (GEST) reported by Oka et al. [7], the ORR of gemcitabine, S-1, and GS combination group was 13.3, 21, and 23.3%, respectively. S-1 was proved not inferior to gemcitabine (9.7 vs. 8.8 months) in OS, while GS (10.1 months) was not superior to gemcitabine monotherapy. Here, in this phase II trial, nab-paclitaxel seemed to be a better partner to S-1.

The high response rate of nab-paclitaxel and S-1in our study is supported by another phase II trial recently reported by Shi et al. [12], in which 60 patients with metastatic pancreatic cancer were enrolled and treated with the same regimen, and the ORR was 50% in the ITT population, the median PFS was 5.6 months, and the median OS was 9.4 months. Furthermore, the combination of nab-paclitaxel with simplified leucovorin and fluorouracil showed good tolerability and certain efficacy (over 50% patients achieved PFS of 4 months) as the first-line chemotherapy for patients with metastatic pancreatic cancer in a recent phase II trial [13]. David et al. [14] conducted a phase II trial of capecitabine plus nab-paclitaxel in patients with metastatic pancreatic adenocarcinoma and observed good tolerability and substantial antitumor efficacy with an ORR of 41.4 and DCR of 76%. Given these preliminary clinical data, the combination of nab-paclitaxel and oral fluoropyrimidine could be an option for pancreatic cancer patients.

In this study, the combination of nab-paclitaxel and S-1 indicated favorable safety profile. The most frequent grade 3/4 adverse effects were neutropenia with an incidence of 27.6% and most of the other adverse events were limited to grade 1/2. Only one patient developed neutropenic febrile. FOLFIRINOX brought survival benefit compared to gemcitabine but led to significantly increased adverse events with an incidence of grade 3/4 neutropenia of 45.7%, fatigue of 23.6%, vomiting of 14.5% and diarrhea of 12.7%, compared to 37% incidence of grade 3/4 neutropenia in MPACT trial, which is higher than the combination of nab-paclitaxel and S-1 in our study.

Findings of this study demonstrated an encouraging efficacy and safety profile of nab-paclitaxel plus S-1 therapy. Further prospective randomized controlled phase III studies are needed to buttress these data. The high antitumor activity may provide a new option in the neoadjuvant setting for patients with locally advanced pancreatic cancer.
